# Alcohol modulation of G-protein-gated inwardly rectifying potassium channels: from binding to therapeutics

**DOI:** 10.3389/fphys.2014.00076

**Published:** 2014-02-25

**Authors:** Karthik Bodhinathan, Paul A. Slesinger

**Affiliations:** ^1^Structural Biology and Peptide Biology Laboratories, The Salk Institute for Biological StudiesLa Jolla, CA, USA; ^2^Department of Neuroscience, Icahn School of Medicine at Mount SinaiNew York, NY, USA

**Keywords:** addiction, alcohol, G proteins, GIRK, potassium channels, Kir3, PIP_2_

## Abstract

Alcohol (ethanol)-induced behaviors may arise from direct interaction of alcohol with discrete protein cavities within brain proteins. Recent structural and biochemical studies have provided new insights into the mechanism of alcohol-dependent activation of G protein-gated inwardly rectifying potassium (GIRK) channels, which regulate neuronal responses in the brain reward circuit. GIRK channels contain an alcohol binding pocket formed at the interface of two adjacent channel subunits. Here, we discuss the physiochemical properties of the alcohol pocket and the roles of G protein βγ subunits and membrane phospholipid PIP_2_ in regulating the alcohol response of GIRK channels. Some of the features of alcohol modulation of GIRK channels may be common to other alcohol-sensitive brain proteins. We discuss the possibility of alcohol-selective therapeutics that block alcohol access to the pocket. Understanding alcohol recognition and modulation of brain proteins is essential for development of therapeutics for alcohol abuse and addiction.

## Introduction

Proper nerve cell communication is critical for brain function and depends on a delicate balance of excitatory and inhibitory signaling. Rapid excitatory signaling is mediated by excitatory ionotropic glutamate receptors, such as the NMDA (N-methyl-D-aspartate) and AMPA (α-amino-3-hydroxy-5-methyl-4-isoxazole propionic acid) receptors. Inhibitory signaling has fast and slow components, which are mediated by fast inhibitory currents through ionotropic GABA_A_-type and glycine receptors and slow inhibitory currents mediated by G protein-coupled receptors (GPCRs) and G-protein-gated inwardly rectifying potassium (GIRK or Kir3) channels. A large family of GPCRs, including those activated by GABA, DA, glutamate, serotonin, acetylcholine and opioids, directly couple to GIRK channels (Ehrengruber et al., 1997; Luscher et al., 1997; Scanziani, 2000; Wiser et al., 2006; Lujan et al., 2009). In addition, GIRK channels are regulated by interaction with neuronal proteins involved in protein trafficking (Lunn et al., 2007; Balana et al., 2013). GIRK channels allow potassium ions to flow into the cell better than out of the cell, a property referred to as “inward rectification.” The small outward current hyperpolarizes the cell's membrane potential, leading to inhibition of neuronal activity. There are four primary neuronal GIRK subunits, GIRK1-GIRK4 (Lesage et al., 1995; Inanobe et al., 1999; Wickman et al., 2000; Luscher and Slesinger, 2010). Alterations in GIRK channel function have been associated with pathophysiology of severe brain disorders like addiction (Hill et al., 2003; Morgan et al., 2003; Labouebe et al., 2007; Kozell et al., 2009), epilepsy (Signorini et al., 1997; Pei et al., 1999; Mazarati et al., 2006), Parkinson's disease and ataxia (Patil et al., 1995; Slesinger et al., 1996; Schein et al., 2005) and Down's syndrome (Siarey et al., 1999; Cooper et al., 2012).

In addition to neurotransmitters that activate GIRK channels via GPCRs, alcohol directly opens GIRK channels at concentrations relevant to human consumption (18 mM ethanol or 0.08% blood alcohol level) (Kobayashi et al., 1999; Lewohl et al., 1999; Aryal et al., 2009). Several laboratories have investigated whether ethanol targets GIRK channels in the brain. First, ethanol enhances GIRK currents in VTA neurons (Federici et al., 2009), where they modify the activity of the VTA neural circuit (Michaeli and Yaka, 2010; Padgett et al., 2012). Second, some of analgesic effects of alcohol were found to involve GIRK channels (Ikeda et al., 2002; Blednov et al., 2003). Third, mice lacking GIRK2 channels consume more ethanol and fail to develop conditioned place preference for ethanol when compared to their wild type littermates (Blednov et al., 2001; Hill et al., 2003). Lastly, quantitative trait loci (QTL) mapping identified the GIRK3 subunit in a 0.44 MB region of chromosome 1 that was associated with withdrawal effects following chronic and acute alcohol exposure (Kozell et al., 2009; Ehlers et al., 2010). Taken together, these studies highlight the significance of GIRK channels in the pathophysiology of alcohol consumption and addiction.

Recently, there has been intense interest in the elucidating the molecular mechanism underlying alcohol dependent modulation of brain proteins (Howard et al., 2011b). In this review, we discuss recent developments in understanding the chemical, physical and structural features of alcohol recognition by GIRK channels and other alcohol-sensitive proteins.

## Alcohol modulation of signaling pathways, proteins, and ion channels

Ethanol affects multiple signaling pathways in the brain, including dopamine (DA) (Theile et al., 2011; Ben Hamida et al., 2012; Li et al., 2012), serotonin (Engel and Allan, 1999; Sung et al., 2000; McBride et al., 2004; Rodd et al., 2010), opioids (Marinelli et al., 2010; Walker et al., 2011), corticosteroids (Vendruscolo et al., 2012), adenosine (Nam et al., 2013), and galanin (Lewis et al., 2004) pathways. Originally, ethanol was hypothesized to interact non-specifically with membrane lipids and consequently, alter the function of integral membrane proteins like ion channels (Harris et al., 2008; Howard et al., 2011b). Indeed, ethanol can modify the activity of some lipid kinases (Tong and Sun, 1996). More recently, it has become clear that ethanol can also modulate ion channels through distinct alcohol binding pockets in the channel protein (Harris et al., 2008; Howard et al., 2011b). Alcohol has been reported to affect several ion channels in the brain. For example, ethanol modulates GABA_A_ (Mihic et al., 1994) glycine receptors (Mihic et al., 1997), Ca^2+^-dependent K^+^ channels (Dopico et al., 1998), and acetylcholine receptors (Cardoso et al., 1999), while ethanol inhibits NMDA receptors (Lovinger et al., 1989). In spite of widely documented effects of alcohol on ion channels and receptors, the demonstration of a direct interaction with an ion channel has been elusive. Unlike canonical ligands that saturate a physical binding site, it has been difficult to show saturation of the alcohol binding pocket because the modulatory effects of ethanol occur in the milliMolar range; saturation would not be evident until ethanol reaches hundreds of milliMolar (>300 mM).

Definitive proof for alcohol interacting directly with ion channels can be obtained from high resolution atomic structures. To date, only a few high-resolution X-ray crystallographic structures exist of ion channels with alcohol bound (Aryal et al., 2009; Howard et al., 2011a; Sauguet et al., 2013). These structures provide a snapshot of the location of alcohol pockets in the channel. However, more detailed experiments are needed to relate the function of alcohol modulation to the physical structure. Nevertheless, these crystal structures have revealed certain fundamental properties of the alcohol pockets. The alcohol pockets are relatively hydrophobic composed of hydrophobic amino acid side chains (e.g., F, L, I) and amino acids that form hydrogen bonds with the hydroxyl in alcohol. Similar pockets have been also described in non-ion channel alcohol targets, such as Drosophila odorant-binding protein LUSH (Kruse et al., 2003), protein kinase C epsilon (Hodge et al., 1999; Newton and Ron, 2007) and alcohol dehydrogenase (Plapp, 2010), suggesting some features of alcohol pockets may be conserved in different types of proteins.

## Structural view of alcohol pocket in GIRK channels

**Figure 1:**
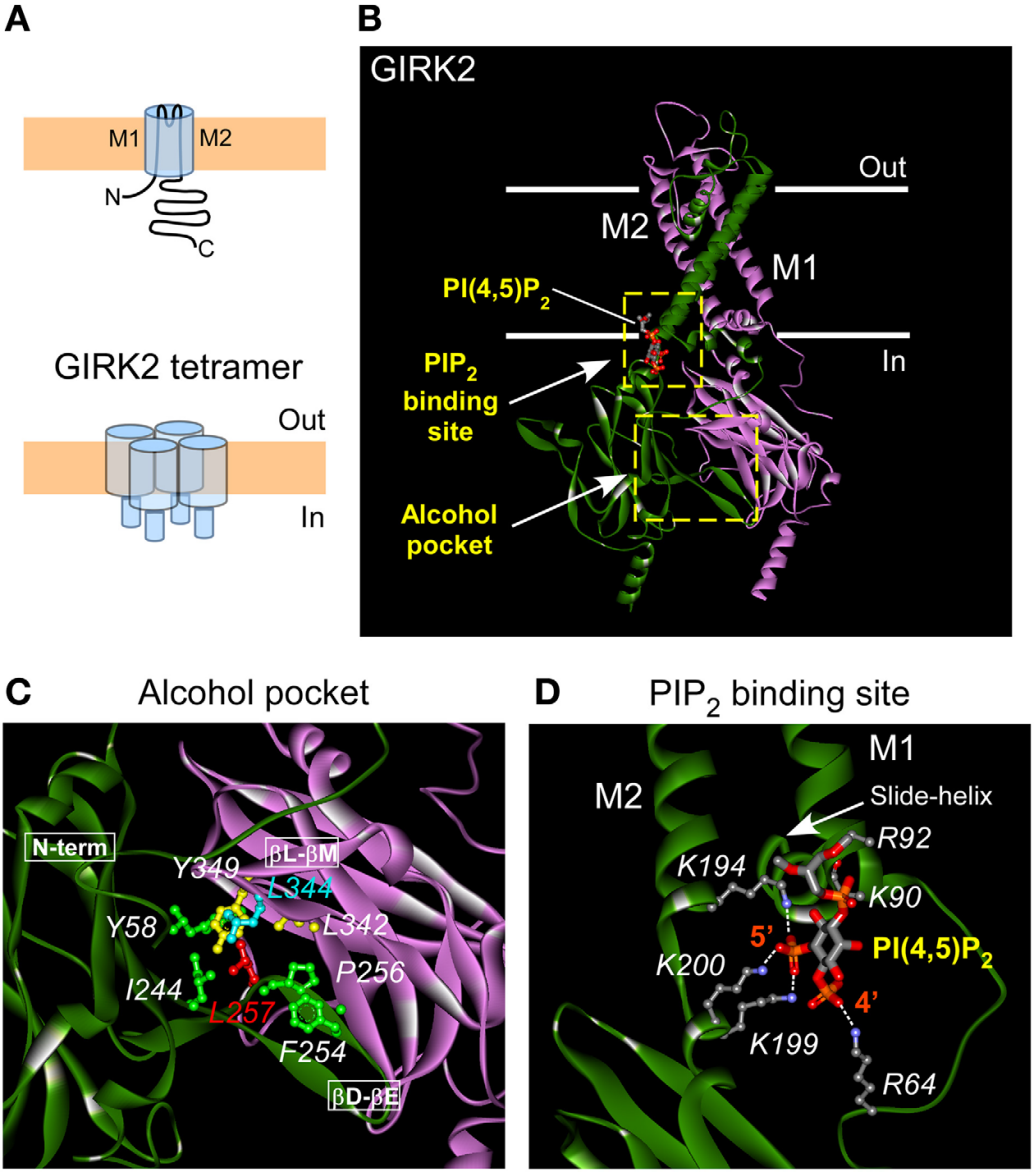
**Structural views of alcohol and PIP2 pockets in GIRK2. (A)** Schematic of GIRK channel monomer (above) and tetramer (below) depicting the N and C terminal regions, and the M1, M2 transmembrane domains. **(B)** Crystal structure (3SYA) of GIRK2 channel (3.6 Å resolution; two of four subunits shown; adapted from Whorton and MacKinnon, 2011) bound to PI(4,5)P_2_/PIP_2_ (indicated by the arrow). The PIP_2_ binding site is located at the interface between transmembrane and cytosolic domains of GIRK channel. Solid white lines indicate the approximate boundary of the membrane lipid bilayer. **(C)** Enlarged view of the alcohol pocket in GIRK2 channel formed by part of N-terminal domain, βD-βE and βL-βM loops from two adjacent subunits (Aryal et al., 2009). Amino acids lining the alcohol pocket are indicated, plus critical Gβγ site L344 on the GIRK2 crystal structure (4KFM, Whorton and MacKinnon, 2013). For a view of alcohol bound to the closely related Kir2.1 channel see Aryal et al., 2009. **(D)** Enlarged view of the PIP_2_ binding site reveals key residues (shown in ball and stick model) involved in binding PIP_2_ and stabilizing the channel's open state. Also indicated are the major points of electrostatic coordination (white dashed line) between the 4′ and 5′ phosphate of PIP_2_ molecule and critical residues (R64, K194, K199, K200) in GIRK2 channel (4SYA).

GIRK channels assemble into heterotetramers of GIRK1/2, GIRK1/3, GIRK1/4, or GIRK2/3 subunits or in some cases homotetramers of GIRK2 subunits (Figure 1A) (Luscher and Slesinger, 2010). The alcohol pocket in GIRK channels is located at the interface between two adjoining GIRK subunits in the cytoplasmic domains (Figures 1B,C) (Aryal et al., 2009). Originally, the alcohol pocket was first identified in Kir2.1 channels with the alcohol MPD (Pegan et al., 2006; Aryal et al., 2009). The alcohol pocket is formed by three prominent structural elements: N-terminal domain and βD-βE loop from one subunit and the βL-βM loop from an adjacent subunit (Pegan et al., 2006; Aryal et al., 2009). Through site-directed mutagenesis of amino acids lining the alcohol pocket in GIRK2, Aryal et al. (2009) demonstrated that the alcohol pocket is the site for alcohol-dependent activation of GIRK2 channels. The hydrophobic pocket appears to be conserved within the family of inwardly rectifying potassium channels and across different species (Supplemental Figure S1). The effect of alcohol in the pocket may not produce the same effect on all Kir channels. For example, Kir2 and Kir1 channels are inhibited or unaffected by alcohols, while GIRK channels are activated (Kobayashi et al., 1999; Lewohl et al., 1999). In addition, a L257W mutation in the GIRK alcohol pocket alters alcohol activation but the analogous mutation in Kir2 channels (L245W) produces little effect on alcohol modulation of Kir2 channels. Furthermore, several other mutations to the Kir2 alcohol pocket (specifically F47W, L232W, and L330W) do not alter modulation by alcohols (Aryal et al., 2009). Many of these analogous mutations in GIRK channels render the channel non-functional, however, precluding determination of alcohol sensitivity.

Recently, the structure of the alcohol pocket in a pentameric ion channel ortholog from the bacterium *Gloeobacter violaceus* (GLIC) (alcohol sensitized variant F14′A) revealed that GLIC shares many features with the alcohol pocket in GIRK channels (Sauguet et al., 2013). Specifically, the ethanol response of both GIRK and GLIC F14′A channels is altered when the volume of the alcohol pockets are altered with side chains of varying volume (Aryal et al., 2009; Sauguet et al., 2013). Another common feature of the pocket is the placement near a structural or gating transition point. In GIRK channels, the alcohol pocket is located at the interface of two adjacent subunits, whereby the βD-βE and βL-βM loops move apart from each other during Gβγ-dependent activation (Finley et al., 2004; Whorton and MacKinnon, 2011). Similarly, the alcohol pocket in GLIC F14′A is located in an intersubunit crevice close to the transmembrane domain (M2), which undergoes a conformational rotation during channel activation (Sauguet et al., 2013). Thus, in both channels, alcohol may serve as an allosteric modulator. Lastly, the chemical interactions of alcohol in the pocket involve both hydrophobic and hydrophilic residues. One notable difference is the physical location of the alcohol pocket. In GLIC, the alcohol pocket is located in the transmembrane domains while the pocket in GIRK channels is situated within the cytoplasmic domains. We anticipate that high resolution structures of alcohol in complex different conformational states of the channel in the future will further clarify the molecular mechanism underlying alcohol modulation of ion channels.

## Chemical and physical properties of alcohol pocket that determine ligand recognition

A remarkable aspect of ethanol's effect on brain function is that this simple chemical compound of only two carbons and a hydroxyl produces long-term behavioral changes in humans. Moreover, ethanol has unusually low potency (mM) and weak selectivity (more than one type of alcohol can achieve similar modulation) for ion channel targets. Recently, Bodhinathan and Slesinger (2013) examined the chemical requirements for activation of GIRK channels using an alcohol-tagging strategy originally described for LGIC channels (Mascia et al., 2000). With a cysteine engineered in the alcohol pocket of GIRK2 (L257C in βD-βE loop), tagging the pocket with both alcohol (hydroxyethyl) and non-alcohol (ethyl or benzyl) like chemical groups led to constitutive channel activation. Thus, the hydroxyl *per se* was not required for chemical activation of GIRK channels, in contrast to modulation with native alcohols. The hydroxyl may be required for stabilizing native alcohols in the pocket through hydrogen bonding. Tagging GIRK2 channels with a hydroxy-benzyl moiety, however, did not activate the channel, indicating that a small increase in side-chain volume was incompatible with channel activation. Importantly, simply attaching alcohol-like compounds to the βD-βE loop was not sufficient for chemical activation, since tagging S246C situated approximately 7 angstroms from L257 was ineffective at promoting channel activation. Thus, proximity to the pocket and hydrophobicity are key features for chemical-dependent activation of GIRK2 channels through the alcohol pocket.

In addition to hydrophobicity, molecular volume is also a major determinant of sensitivity to alcohol modulation. Based on the estimated volume of different alcohols that activate GIRK channels and the volume of amino acid side-chains that line the pocket, the GIRK alcohol pocket is estimated to be ~312 Å^3^ (Bodhinathan and Slesinger, 2013). This volume could optimally accommodate two or three ethanol molecules. By comparison, the alcohol pocket of the GLIC F14′A channel has been estimated to be ~335 Å^3^ (Howard et al., 2011a), but contained only one bromo-ethanol compound. Future high-resolution crystal structures of alcohol bound to the GIRK alcohol pocket will be needed to determine the precise stoichiometry of ethanol and its stereospecific arrangement within the pocket.

In summary, the emerging view of the physiochemical properties of the alcohol pocket suggests that ligand occupancy and modulation through these pockets are determined by the hydrophobicity and volume. In addition, the location of the alcohol pockets at the interface of channel subunits (e.g., GIRK and GLIC) reveals a fundamental topographical design that makes it accessible to alcohol and facilitates intersubunit conformational changes that underlie channel opening. These features enable the alcohol pockets to act as critical modulatory sites for these channels.

## Convergence of GIRK channel regulators with alcohol-dependent activation

The primary pathway for GIRK channel activation occurs through stimulation of GPCRs that couple to pertussis-toxin sensitive G proteins (Gi/o), which in turn directly activate GIRK channel via G protein Gβγ subunits (Logothetis et al., 1987; Reuveny et al., 1994; Wickman et al., 1994; Huang et al., 1995; Kunkel and Peralta, 1995). Mutagenesis and biochemical Gβγ binding experiments implicated several regions in the cytoplasmic domains involved in Gβγ activation, with a particular emphasis on the involvement of a Leucine in the βL-βM loop (L344 in GIRK2, L333 in GIRK1) (Huang et al., 1995, 1997; He et al., 1999; Ivanina et al., 2003; Finley et al., 2004). Whorton and MacKinnon (2013) recently solved the crystal structure of a GIRK2-Gβγ complex and confirmed that L344 interacts directly with the Gβ subunit. Leu55 on Gβ forms hydrogen bonds with L344 as well as with several other sites near the alcohol pocket (F254, P256, L342, and Y349). Similarly, taking a computational approach, Mahajan et al. (2013) recently pinpointed an interaction between L55 in Gβ and L333 in GIRK1, demonstrating that a disulfide can form between L55C and L333C and lead to sustained activation (Mahajan et al., 2013). According to this model, Gβγ binds to the GIRK channel at the cleft formed by the βD-βE and βL-βM loop from adjacent GIRK subunits, stabilizing the open conformation of the G loop gate that precedes channel opening and ion permeation. Remarkably, the region of Gβγ interaction overlaps completely with the alcohol pocket (Bodhinathan and Slesinger, 2013). GIRK channels are also regulated (but not activated) by Gα subunits (Ivanina et al., 2004; Clancy et al., 2005; Rubinstein et al., 2007). Interestingly, NMR experiments suggest a different region of the cytoplasmic domain is involved (Mase et al., 2012).

Previous studies suggested that alcohol activation occurs independently from receptor-dependent activation (Kobayashi et al., 1999; Lewohl et al., 1999). Using an alcohol tagging strategy with GIRK2, Bodhinathan and Slesinger (2013) found that MTS-HE modification of the Gβγ L344 site reduced basal GIRK2 current, while modification of L257 increased the basal GIRK2 current. Furthermore, varying the levels of Gβγ subunits consistently altered the rate of MTS-HE-dependent inhibition of L344C but had little effect on modification of L257C. Thus, in spite of the considerable overlap in the alcohol and Gβγ binding sites, activation by MTS-HE does not seem to be influenced by alterations in the Gβγ levels. Taken together, these results support a model where association between Gβγ and GIRK2 L344 in the βL-βM loop, similar to L333 in GIRK1 (Mahajan et al., 2013), precedes alcohol-mediated activation in the pocket.

A regulator that is common to all inwardly rectifying K^+^ channels is the membrane-bound phospholipid-phosphatidylinositol 4,5-bisphosphate (PIP_2_) (Huang et al., 1998; Zhang et al., 1999). PIP_2_ is required for the constitutive basal activity of inward rectifiers like Kir1, Kir2 and Kir3 (GIRK) channels. PIP_2_ association also underlies agonist-dependent activation of GIRK channels (Huang et al., 1998; Zhang et al., 1999; Xiao et al., 2003). An examination of the relative affinity for PIP_2_ indicated that GIRK channels interact weakly with PIP_2_ and Gβγ-dependent activation increases the affinity for PIP_2_ (Huang et al., 1998; Zhang et al., 1999). High-resolution structural studies indicate that the 5' phosphate in PIP_2_ is critical for binding to GIRK at the membrane-cytosol interface (Figure 1D) (Whorton and MacKinnon, 2011). Like Gβγ-dependent activation, alcohol-dependent activation also depends on PIP_2_ interaction with the channel. Alcohol fails to activate GIRK channel in cells where PIP_2_ levels are depleted using a voltage-activated phosphatase (Dr-VSP) that removes the 5′ phosphate (Bodhinathan and Slesinger, 2013). Moreover, presence of alcohol in the pocket slows down that rate of PIP_2_ dissociation from the channel, suggesting an increase the relative affinity between PIP_2_ and the GIRK channel. Thus, structural changes in the PIP_2_ binding interface in GIRK channels is a critical gating step for two distinct pathways for activating GIRK channels, slower Gβγ-dependent activation and rapid alcohol-dependent activation.

**Figure 2:**
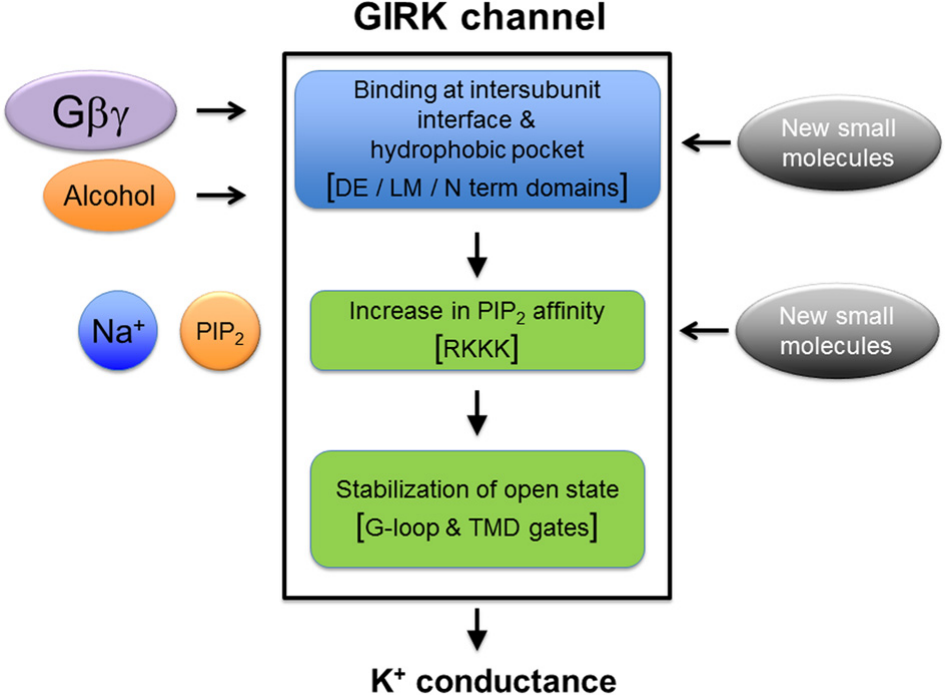
**Working model of GIRK channel gating.** Alcohol binds directly to the alcohol pocket and Gβγ binds very close to the alcohol pocket. The binding leads to multi-domain conformation changes that culminate in stabilization of the PIP_2_-bound open state of GIRK channel; involving increase in PIP_2_-GIRK affinity (Bodhinathan and Slesinger, 2013) and possibly involving structural changes to the PIP_2_ binding site described in Figure 1. In addition, small molecule modulators of GIRK channel can possibly interact with the alcohol binding site, or may directly stabilize PIP_2_-bound open state of the GIRK channels. These events lead to the movement and opening of the G loop and the transmembrane gates, the final step of activation leading to K^+^ ion permeation through the GIRK channel pore.

Alcohol may facilitate a “sliding” movement of the two adjacent GIRK subunits, increasing the affinity for PIP_2_, and inducing movement of the channel's G loop and transmembrane gates to allow ion permeation (Pegan et al., 2005; Whorton and MacKinnon, 2011, 2013; Mahajan et al., 2013). Increasing the hydrophobicity of the pocket, either by chemical-tagging with an alcohol-like short-chain molecule or by native alcohol itself, lowers the free energy barrier (ΔG) for channel opening (Figure 2), similar to alcohol-sensitive ligand-gated ion channels (LGICs) (Mascia et al., 2000). Alcohol binding to the pocket may produce weak van der Waal and hydrogen bond interactions with several residues in the βD-βE and βL-βM loops that line the pocket of GIRK channels and stabilize an open state, through increasing the PIP_2_-GIRK affinity. In the alcohol-sensitized GLIC, the B-factor is decreased in the presence of ethanol, suggesting that alcohol also stabilizes an open state (Sauguet et al., 2013). An attractive feature of an allosteric model for gating is that it is compatible with (1). low affinity of GIRK channels for alcohol, (2). low binding energy associated with alcohol-dependent activation of GIRK, and (3). apparent lack of chemical specificity for alcohols that can be accommodated within the GIRK alcohol pocket (Harris et al., 2008). How it is that alcohol- and Gβγ-dependent activation mechanisms are independent yet have partially overlapping binding sites remains an important question for future studies.

In fact, the involvement of changes in affinity between PIP_2_ and GIRK channels is emerging as a common theme in the activation of GIRK channels by alcohol (Bodhinathan and Slesinger, 2013), Gβγ (Huang et al., 1998; Whorton and MacKinnon, 2013) and Na^+^ (Ho and Murrell-Lagnado, 1999; Petit-Jacques et al., 1999; Inanobe et al., 2010; Whorton and MacKinnon, 2011). Based on this model of convergence at the PIP_2_ binding site for these distinct activators, we can highlight certain unanswered points. Alcohol binding at the cytoplasmic domain could structurally re-arrange the PIP_2_ binding site in a manner that increases the physical retention of PIP_2_ at this site (Xiao et al., 2003; Whorton and MacKinnon, 2011). One question that remains unanswered is whether the physical rearrangements in the alcohol pocket are inextricably linked to changes in PIP_2_ binding. Based on the current results, it is unlikely a GIRK mutant will be identified that is deficient in PIP_2_ binding but has normal alcohol activation. A related question is whether a point mutation will be identified that creates an alcohol resistant GIRK channel that retains Gβγ activation. Lastly, it is unknown what determines the subtle differences in alcohol activation of GIRK channels composed of different subunits (Kobayashi et al., 1999; Lewohl et al., 1999). Thus, any therapeutic strategy to treat alcohol addiction and abuse will need to take into account these remaining questions.

## Clinical implications

Currently, there are no adequate treatments for alcohol dependence and addiction. Traditional approaches to treat alcohol abuse problems have targeted the stress and anxiety pathways in the brain to ameliorate alcohol craving and manage painful withdrawal symptoms (Silberman et al., 2009; Zorrilla and Koob, 2010; Pastor et al., 2011). Recently, several therapeutic strategies are currently approved or in clinical trials for treating alcoholism. In 2012, Baclofen was approved as treatment for alcoholism by the French agency AFSSAPS. In U.S., based on recent advances in clinical research (Ameisen, 2005; Addolorato et al., 2011), NIAAA is finishing Phase-2 clinical trials testing Baclofen as treatment for alcoholism (ClinicalTrials.gov Identifier: NCT01751386). Baclofen is an agonist for GABA_B_ receptors, which activate GIRK channels, inhibit voltage-gated calcium channels, and alter cAMP levels. However, Baclofen, originally developed to treat muscular spasticity (Penn and Kroin, 1985), produces numerous undesirable side-effects like headache, sleepiness, exhaustion, vertigo, nausea, and insomnia (Addolorato et al., 2011).

Another strategy for treating alcoholism could be to antagonize the direct action of alcohol in the brain through alcohol-selective therapeutics. Understanding the chemical nature of the GIRK alcohol pocket has revealed unique chemical rules that are associated with channel activation. The chemical rules of hydrophobicity and size of the ligand occupying the pocket, have significant implications for the development of chemical therapeutics that can occupy the pocket and selectively prevent alcohol access to GIRK channels. It may be possible to design smart chemicals that do not engage the channel activation mechanism through the pocket but selectively block access to alcohol. Interestingly, numerous synthetic ligands have been reported to modulate GIRK channel function, including antidepressants (Kobayashi et al., 2004, 2011; Hamasaki et al., 2013), antipsychotics (Kobayashi et al., 2000; Heusler et al., 2011), and anesthetics (Slesinger, 2001; Yamakura et al., 2001; Zhou et al., 2001; Styer et al., 2010). Recently, a small compound was described that selectively activates heterotetramers containing the GIRK1 subunit and exhibits anti-epileptic properties (Kaufmann et al., 2013). It is not known where this compound acts, but it offers an opportunity to discover possible antagonists for ethanol-dependent activation.

## Future challenges

To fully describe the universal properties of alcohol pockets, more high resolution crystal structures of ion channels bound to alcohol are needed. Future studies exploring the link between changes in alcohol pocket chemistry and ion channel structure will be helpful for developing novel anti-addiction therapeutics that carry minimal dependency and high degree of selectivity. Whether there are differences in ethanol sensitivity amongst different GIRK subunits is poorly understood. Of particular note, GIRK2/3 channels are exclusively expressed in VTA DA neurons (Cruz et al., 2004) and exhibit reduced sensitivity to Gβγ (Jelacic et al., 2000).

Although these studies implicate the GIRK channel in alcohol binding, it remains unknown whether altering the GIRK channel composition or alcohol pocket chemistry will yield “alcohol-resistant” mutants. For example, future studies will need to identify mutations with the alcohol pocket of GIRK channels from human or closely related rat and mouse GIRK channels, which exhibit alcohol-deficient response but normal G protein gating. Such GIRK mutants can be used to create novel knock-in animals that do not develop alcohol-related behavioral changes or alcohol addiction. Furthermore, it is predicted that such knock-in animals will have fewer phenotypic side effects due to unaltered G protein response. On the other hand, altering the alcohol pocket chemistry in GIRK channels can enhance ethanol response, which would be an unwanted side-effect. This finding highlights the intricate chemical and physical nature of the alcohol pocket in GIRK channel.

## Author contributions

Karthik Bodhinathan and Paul A. Slesinger co-wrote the manuscript.

## Conflict of interest statement

The authors declare that the research was conducted in the absence of any commercial or financial relationships that could be construed as a potential conflict of interest.

## Supplementary material

Supplemental Figure S1 **Conservation of amino acids in alcohol pocket across species**. Clustal Omega (EMBL-EBI) alignment of GIRK2 (Kir3.2/KCNJ6) sequences from different species reveals a high degree of conservation in the domains forming the alcohol pocket: N-terminal domain (pink), βD-βE loop (green) and βL-βM loop (blue). Leucine 257 (bold), which plays a critical role in alcohol activation, is conserved across multiple species. The respective NCBI sequence ID is also indicated.
