# Supplementary material for: Alcohol modulation of G-protein-gated inwardly rectifying potassium channels: from binding to therapeutics
Source: Front Physiol. 2014 Feb 25;5:76. doi: 10.3389/fphys.2014.00076 (PMC3933770; doi:10.3389/fphys.2014.00076)
Supplement: Supplemental Figure S1 — Conservation of amino acids in alcohol pocket across species. Clustal Omega (EMBL-EBI) alignment of GIRK2 (Kir3.2/KCNJ6) sequences from different species reveals a high degree of conservation in the domains forming the alcohol pocket: N-terminal domain (pink), βD-βE loop (green) and βL-βM loop (blue). Leucine 257 (bold), which plays a critical role in alcohol activation, is conserved across multiple species. The respective NCBI sequence ID is also indicated. [file Presentation1.PDF]

GIRK2 (Kir3.2/KCNJ6)

	N-terminus	βD-βE	βL-βM	NCBI ID
Pigeon	KRKIQRYVRKDGKCNV	KLIKSKQTKEGEFIPL	FTPVLTLEDGFYEVDY	EMC90378
Rat	KRKIQRYVRKDGKCNV	KLIKSKQTSEGEFIPL	FTPVLTLEDGFYEVDY	NP_037324
Mouse	KRKIQRYVRKDGKCNV	KLIKSKQTSEGEFIPL	FTPVLTLEDGFYEVDY	NP_001020755
Squirrel	KRKIQRYVRKDGKCNV	KLIKSKQTSEGEFIPL	FTPVLTLEDGFYEVDY	XP_005323544
Horse	KRKIQRYVRKDGKCNV	KLIKSKQTSEGEFIPL	FTPVLTLEDGFYEVDY	XP_005606207
Human	KRKIQRYVRKDGKCNV	KLIKSKQTSEGEFIPL	FTPVLTLEDGFYEVDY	NP_002231
Orangutan	KRKIQRYVRKDGKCNV	KLIKSKQTSEGEFIPL	FTPVLTLEDGFYEVDY	NP_001127100
Manatee	KRKIQRYVRKDGKCNV	KLIKSKQTSEGEFIPL	FTPVLTLEDGFYEVDY	XP_004389494
Sheep	KRKIQRYVRKDGKCNV	KLIKSKQTSEGEFIPL	FTPVLTLEDGFYEVDY	XP_004003459
whale	KRKIQRYVRKDGKCNV	KLIKSKQTSEGEFIPL	FTPVLTLEDGFYEVDY	XP_004264606
	****************	********.*******	****************	
		L257		

Supplemental Fig S1
